# Implication of miR-126 and miR-139-5p in Plasmacytoid Dendritic Cell Dysregulation in Systemic Sclerosis

**DOI:** 10.3390/jcm10030491

**Published:** 2021-01-30

**Authors:** Eleni Chouri, Maojie Wang, Maarten R. Hillen, Chiara Angiolilli, Sandra C. Silva-Cardoso, Catharina G. K. Wichers, Maarten van der Kroef, Cornelis P. J. Bekker, Marta Cossu, Lenny van Bon, Alsya J. Affandi, Tiago Carvalheiro, Aridaman Pandit, Joel A. G. van Roon, Lorenzo Beretta, Boudewijn M. T. Burgering, Timothy R. D. J. Radstake, Marzia Rossato

**Affiliations:** 1 Center of Translational Immunology, University Medical Center Utrecht, Utrecht University, 3584 CX Utrecht, The Netherlands; eleni.chouri@gmail.com (E.C.); hillen.maarten@gmail.com (M.R.H.); C.Angiolilli@umcutrecht.nl (C.A.); sandracsc9@gmail.com (S.C.S.-C.); R.Wichers@umcutrecht.nl (C.G.K.W.); Maartenkroef1988@gmail.com (M.v.d.K.); C.P.J.Bekker-2@umcutrecht.nl (C.P.J.B.); marta.cossu@gmail.com (M.C.); lennygeurts@gmail.com (L.v.B.); a.affandi@amsterdamumc.nl (A.J.A.); t.ferreiracarvalheiro@umcutrecht.nl (T.C.); A.Pandit@umcutrecht.nl (A.P.); J.vanRoon@umcutrecht.nl (J.A.G.v.R.); tradstake73@gmail.com (T.R.D.J.R.); 2 Department of Rheumatology and Clinical Immunology, University Medical Center Utrecht, Utrecht University, 3584 CX Utrecht, The Netherlands; 3 Department of Molecular Cancer Research, Center Molecular Medicine, Oncode Institute, University Medical Center Utrecht, Utrecht University, 3584 CX Utrecht, The Netherlands; M.Wang-2@umcutrecht.nl (M.W.); B.M.T.Burgering@umcutrecht.nl (B.M.T.B.); 4 Scleroderma Unit, Referral Center for Systemic Autoimmune Diseases, Fondazione IRCCS Ca’ Granda Ospedale Maggiore Policlinico di Milano, 20122 Milan, Italy; lorberimm@hotmail.com; 5 Department of Biotechnology, University of Verona, 37134 Verona, Italy

**Keywords:** plasmacytoid dendritic cells, microRNAs (miRNAs), systemic sclerosis

## Abstract

Compelling evidence shows the involvement of plasmacytoid dendritic cells (pDCs) in systemic sclerosis (SSc) pathogenesis. This study investigated whether microRNAs (miRNAs) are involved in the dysregulation of pDCs in SSc patients already at early stages. RNA from circulating pDCs was isolated from two independent cohorts of SSc patients with different disease phenotypes, and individuals with Raynaud’s phenomenon, for microRNA profiling and RNA-sequencing analysis. Proteomic analysis was exploited to identify novel direct miRNA targets at the protein level. Twelve and fifteen miRNAs were differentially expressed in at least one group of patients compared to healthy controls in discovery cohort I and II, respectively. Of note, miR-126 and miR-139-5p were upregulated in both preclinical and definite SSc patients and correlated with the expression of type I interferon (IFN)-responsive genes. Toll-like receptor 9 (TLR9) stimulation of healthy pDCs upregulated the expression of both miRNAs, similarly to what was observed in patients. The proteomic analysis identified USP24 as a novel target of miR-139-5p. The expression level of USP24 was inversely correlated with miR-139-5p expression in SSc patients and induced by TLR9 stimulation in healthy pDCs. These findings demonstrated that the miRNA profile is altered in pDCs of SSc patients already at early stages of the disease and indicate their potential contribution to pDC activation observed in patients.

## 1. Introduction

Systemic sclerosis (SSc) is a complex autoimmune disorder characterized by connective tissue fibrosis in the skin and internal organs. The exact molecular pathogenesis of the disease is poorly understood; however, profound vascular dysregulation and immune activation precede fibrosis [1,2]. Humoral and cellular immunological abnormalities, including immune cell infiltration in the affected tissues and secretion of immune mediators, are evident in SSc [1]. In addition, the presence of inflammatory hallmarks, including the type I interferon (IFN) signature and the characteristic autoantibody profile fingerprint, further emphasizes the implication of immune dysregulation in these patients [1,3,4].

Recent insights have underlined the role of plasmacytoid dendritic cells (pDCs) in SSc pathogenesis [5,6]. PDCs are the major interferon producing cells, suggesting their role in perpetuating the type I IFN signature characterizing these patients. Consistently, Kim et al. showed that serum from SSc patients, containing autoantibodies, induced pDC activation and interferon-α (IFN-α) production from healthy cells [7] via the uptake of immune complexes through the Fc-gamma (Fcγ) receptor and the interaction with Toll-like receptors (TLR) [7,8]. In addition, pDCs from SSc patients secrete high levels of Chemokine (C-X-C Motif) Ligand 4 (CXCL4), and the increased amount of circulating CXCL4 correlates with the extent of skin fibrosis, as measured by the modified Rodman skin score (mRSS), and lung fibrosis [5]. Indeed, pDCs infiltrate the skin of SSc patients, where they release IFN-α and CXCL4 [5,6]. Furthermore, pDCs of SSc patients show aberrant TLR8 signaling [6] and secrete higher IFN-α upon TLR stimulation as compared to healthy cells [5]. Consistently, the depletion of pDCs reduced skin fibrosis in a mouse model of scleroderma with established disease, and their depletion could prevent disease [6].

However, the underlying mechanism that mediates pDC dysregulation in SSc remains elusive. Recently, it was demonstrated that microRNA-618 (miR-618) is upregulated in pDCs of lcSSc and dcSSc patients, and it is correlated with altered pDC differentiation and activation [9]. Despite the pivotal role of microRNAs (miRNAs) in immune system activation, as well as in the pathogenesis of several autoimmune diseases [10], it is not fully clarified whether the alteration of this epigenetic mechanism is implicated in the immune activation preceding the onset of fibrosis in SSc.

Under the hypothesis that miRNAs underlie the dysregulation of pDC in the earliest clinical phases preceding definite SSc, we investigated the expression of 758 miRNAs using a screening approach by qPCR-based OpenArray platform in two separate cohorts. The first cohort included individuals with Raynaud’s phenomenon (RP), patients with early preclinical symptoms of the disease, as well as non-skin fibrotic SSc patients, while the second cohort contained both non-skin fibrotic SSc and SSc patients with fibrotic skin features. Here, we identified two miRNAs (miR-126 and miR-139-5p) that were increased in both early preclinical and definite SSc patients compared to the healthy controls. Furthermore, we performed a combined-multiple layer analysis, functional studies, and stable isotope labeling of amino acids (SILAC) followed by proteomic analysis to investigate the role of these miRNAs in the immune alteration in SSc pathogenesis.

## 2. Material and Methods

### 2.1. Patients

Blood was collected from 72 patients and 26 healthy controls (HC) in the Scleroderma Unit of the Fondazione IRCCS Policlinico of Milan in Italy (discovery cohort I) and the University Medical Center Utrecht (discovery cohort II and validation cohort) in the Netherlands. The study has been conducted on consecutive SSc patients presenting with the desired phenotypes and recruited in the time frame of the study. All patients signed written informed consent before participation, which was approved by the local ethic committees. Patients with Raynaud’s phenomenon were included in this study (RP). Early SSc (eaSSc) patients were classified according to Leroy and Medsger [11]. Definite SSc patients fulfilling the classification criteria [12] without manifesting skin fibrosis were defined as non-cutaneous SSc (ncSSc); fibrotic SSc patients were stratified to limited cutaneous (lcSSc) or diffuse cutaneous SSc (dcSSc) according to the extent of skin involvement [13]. 

Three separate cohorts were recruited for the purpose of this study. Demographics and clinical characteristics of the patients included in discovery cohort I and II and the validation cohort are shown in Table 1.

### 2.2. Blood Collection, Plasmacytoid Dendritic Cell Isolation, and RNA Extraction

Blood was collected in Lithium–Heparin tubes (BD Vacutainer, Franklin Lakes, NJ, USA). Circulating pDCs were isolated from peripheral blood by positive selection using CD304 (BDCA-4) magnetic beads on AutoMacs Pro (Milteny Biotec, Bergisch Gladbach, Germany) according to the manufacturer’s protocol. PDCs were lysed in RLTplus (Qiagen, Hilden, Germany) with b-mercaptoethanol. RNA from pDCs was isolated using the Allprep Universal Kit (Qiagen), according to the manufacturer’s instructions. RNA quantification was performed on Qubit 2.0 fluorimeter (Invitrogen, Thermo Fisher Scientific, Waltham, MA, USA) using the Qubit RNA HS Assay Kit (Invitrogen). RNA integrity (RNA integrity number ≥8.0) was assessed using RNA 6000 Nano Kit (Agilent Technologies, Santa Clara, CA, USA).

### 2.3. MiRNA Profiling 

MiRNA profiling was conducted using the Taqman OpenArray microRNA platform (Applied Biosystems, Thermo Fisher Scientific, Waltham, MA, USA) according to the manufacturer’s instructions with minor adjustments. Then, 10 ng of RNA was retrotranscribed using Megaplex Primer Pools (Applied Biosystems), and cDNA was pre-amplified using Megaplex PreAmp Primer pools (Applied Biosystems) as previously described [14]. The miRNA profiling was conducted on QuantStudio 12 k flex Real-Time PCR system (Applied Biosystems). ExpressionSuite Software (Applied Biosystems) was used to analyze the data by applying the relative quantification method. Low expressed miRNAs, with Crt (relative threshold cycle) higher than 27 or low amplification score, were excluded from the analysis. The expression of each miRNA was normalized globally to the mean expression of all the expressed miRNAs. The relative fold change (FC) of each miRNA was normalized to the mean of the control group (healthy controls).

### 2.4. MiRNA, pDC Culture, and Gene Expression Analysis

The validation of the miRNA expression was performed using a custom OpenArray microRNA platform (Applied Biosystems) on the QuantStudio 12k flex System, following the manufacturer’s instructions. MiRNA expression values were calculated according to the comparative threshold cycle method (Ct) after normalization to the RNU48, snRNA-U6, has-miR-17, and has-miR-191, using Thermo Fisher Scientific cloud software (https://www.thermofisher.com/account-center/cloud-signin-identifier.html).

Healthy control pDCs were isolated from PBMCs as described above. PDCs were cultured in Roswell Park Memorial Institute Medium (RPMI Medium 1640, Gibco, Thermo Fischer Scientific) supplemented with Glutamax (Gibco, Thermo Fischer Scientific) containing 10% heat inactivated fetal bovine serum (FBS) (Biowest, Nuaillé, France). Then, 100,000 cells were seeded in 96-well round plates in the culture medium supplemented with 10 ng/mL IL-3 (ImmunoTools, Friesoythe, Germany). After resting the cells for 2 h, pDCs were either left untreated or stimulated with 10 µg/mL TLR7/8 (R848, Invivogen, San Diego, CA, USA) agonists and 1 µM TLR9 ligand (CpG-C, Invivogen) agonists for 24 h. Cells were lysed in RLT plus supplemented with b-mercaptoethanol. The expression of miR-126 (hsa-miR-126, ID: 002228) and miR-139-5p (hsa-miR-139-5p, ID: 002289) was assessed by a single Taqman Real-Time qPCR in Quantstudio 12k Flex System (Applied Biosystems). MiRNA expression was normalized to U6 (U6 snRNA, ID: 001973) levels, and the relative expression (FC) was normalized to the control untreated group (FC = 2^−ΔΔCt^, ΔΔCt = ΔCtsample – ΔCtreference). 

The expression of protein-coding genes was assessed by Real-Time PCR. RNA was retro-transcribed using SuperScript (Invitrogen) and qPCR was performed on a QuantStudio 12k flex System using SybrSelect mastermix (Applied BioSystems). Data were normalized to the expression of *GUSB* and analyzed as described above for the miRNAs.

### 2.5. RNA Sequencing 

Whole transcriptome analysis was performed by RNA-seq technology at the Beijing Genomics Institute (BGI, Shenzhen, China). RNAseq libraries were prepared from 100 ng total RNA using the TruSeq Stranded kit (Illumina, San Diego, CA, USA) after poly (A) capture, according to the manufacturer’s instructions. Then, libraries were pooled at equimolar concentration and sequenced on an HiSeq 2000 sequencer (Illumina) by applying standard manufacturer protocols. About 20 million paired-end (100 bp) reads were generated for each sample. The samples were quality checked using FastQC and were aligned to the reference human genome (GRCh38 built 79) using STAR aligner [15]. Read counts per gene were calculated using HTSeq-count [16]. Differential gene expression analysis between healthy donors and SSc subsets was performed with DESeq2 [17], and the read counts were normalized using variance stabilizing transformation [18] to obtain normalized read counts. 

### 2.6. Proteomics (SILAC)

Stable isotope labelling of amino acids in cell culture (SILAC) was performed in human cell line HEK 293T cells. Cells were pre-cultured in Dulbeccos Modified Eagle Medium (DMEM, Gibco, Thermo Fischer Scientific) supplemented with 10% FBS plus heavy (^13^C,^15^N)- or light (^12^C,^14^N)-labeled Arg+Lys for at least 14 days to achieve steady-state labeling, before transfection with 30 nM of miRNA mimic (Ambion, Thermo Fischer Scientific) or negative control mimic (Ambion) by using Lipofectamin RNAimax (Invitrogen) for 48 h. Cells were washed twice by PBS and harvested by adding lysis buffer (8 M Urea, 1 M ammonium bicarbonate, 10 nM tris (2-carboxyethyl) phosphine, 40 nM chloroacetamide). Lysates were incubated at 95 °C for 5 min, sonicated, and diluted to 2M Urea with 1 M ABC. Proteins were quantified using bicinchoninic acid (BCA) protein assay, and 150 μg each of miRNA- or control-treated cell lysates were mixed, followed by overnight proteolysis with 2% (w/w) trypsin at 37 °C. Peptides in each sample were separated into 20 fractions by UPLC (UltiMate-3000 system, Thermo Fischer Scientific). Peptides in fraction were collected and further recovered through the C18 filter by centrifugation, dried by speed vacuumed centrifugation, and stored at −80 °C. MS/MS was performed using an Orbitrap Fusion system (Orbitrap Fusion Tribrid Mass Spectrometer, Thermo Fisher Scientific). Raw data were processed using MaxQuant and Perseus was used for further data analysis. 

### 2.7. Statistical Analysis

MicroRNA profiling data were analyzed on ExpressionSuite software (Applied Biosystems), applying an independent *t*-test on the comparative Ct method (ΔΔCt), and differences were considered significant at the level of *p* < 0.05 (uncorrected *p*-value). GraphPad Prism v6.0 Software (GraphPad Software, San Diego, CA, USA) or IBM SPSS 21 Software (IBM, Armonk, NY, USA) were used to assess the Wilcoxon signed-rank test or compute the correlation analysis Spearman’s rho. *p*-values of <0.05 were considered significant. 

The miRWalk 2.0 database (http://zmf.umm.uni-heidelberg.de/apps/zmf/mirwalk2/custom.html) was used to retrieve miRNA predicted targets from 5 different prediction software (Miranda, miRDB, Pictar2, PITA, RNA22, Targetscan) and also to retrieve the validated targets. The ToppGene Suite website (https://toppgene.cchmc.org/enrichment.jsp) was used for the pathway enrichment analysis. Venn’s diagrams were performed on http://bioinfogp.cnb.csic.es/tools/venny/. Heat maps were performed in heatmaper using hierarchical clustering with average linkage (http://heatmapper.ca/).

## 3. Results

### 3.1. Experimental Design

**Figure 1:**
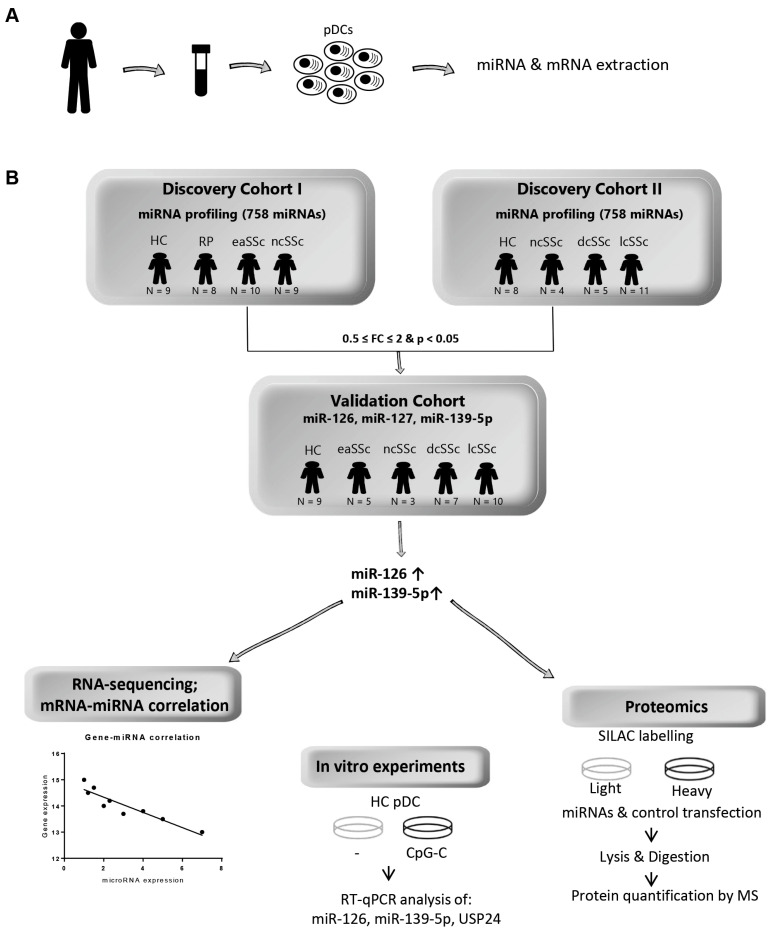
Schematic design of the study. (**A**) Peripheral blood plasmacytoid dendritic cells (pDCs) were isolated from three independent cohorts of SSc patients (*n* = 72) and matched healthy donors (*n* = 26), and RNA was extracted for further miRNA analysis and RNA sequencing. (**B**) A qPCR-based array was used to determine the expression of 758 miRNAs in discovery cohort I and II, while the transcriptome profile of the same cells was determined using RNAsequencing. Two independent experiments were performed for both miRNA screening and RNAsequencing. MicroRNA profiling was analyzed using ExpressionSuite Software (independent t-test, uncorrected *p*-value < 0.05). For RNA sequencing, differential gene expression analysis (adjusted *p*-value < 0.05) was performed with DESeq2, and the read counts were normalized using variance stabilizing transformation to obtain normalized read counts. Stable isotope labeling of amino acids (SILAC) followed by proteomic analysis was used to assess the protein targets of miR-126 and miR-139-5p. These miRNAs were further correlated with RNA sequencing data. HC, healthy control; RT-qPCR, Real-Time quantitative PCR; MS, mass spectrometry.

In order to expand the knowledge on the role of miRNA-mediated mechanisms in the dysregulation of the immune system that may precede SSc onset, we isolated peripheral blood pDCs from two independent discovery cohorts of SSc patients and healthy donors recruited in consecutive manner (Table 1). Discovery cohort I included nine healthy donors and 27 Italian patients with symptoms representative of the earliest preclinical phase of SSc, i.e., having Raynaud’s phenomenon (RP), or early SSc (eaSSc), but also definite SSc without skin fibrosis (ncSSc). Although RP is not a specific symptom for SSc, RP patients were included, because this is first manifestation of SSc in 95% of cases, preceding the onset of SSc by years. In addition, we have previously shown that RP patients display an intermediate molecular feature between healthy donors and eaSSc, namely a trend in IFN-signature upregulation [4]. Discovery cohort II included Dutch definite SSc patients with ncSSc, lcSSc, and dcSSc phenotypes (*n* = 20), as well as healthy controls (*n* = 8). The two cohorts were analyzed in parallel to cover the whole set of SSc subsets, but results were kept separated to avoid the different ancestry (Tuscan and Central European, respectively) confounding the analysis. A qPCR-based array was used to determine the expression of 758 miRNAs, while the transcriptome profile of the same cells was analyzed using RNA-sequencing (Figure 1). Provided that we used loose cut-offs (i.e., no correction for multiple testing) in the discovery phase to decrease chances of excluding potentially relevant targets, we narrowed down the selection to miRNA differentially expressed in multiple comparisons and validated them in a third independent cohort (Validation cohort, *n* = 34). For the replicated miRNAs, novel target genes were identified using stable isotope labeling of amino acids (SILAC) followed by proteomic analysis, and the inverse correlation of miRNAs with putative targets was assessed in order to identify the potential contribution of these miRNAs to SSc-pDC alterations (Figure 1B).

### 3.2. The Profile of miRNAs Is Altered in pDCs from Patients with Early and Definite SSc

**Figure 2:**
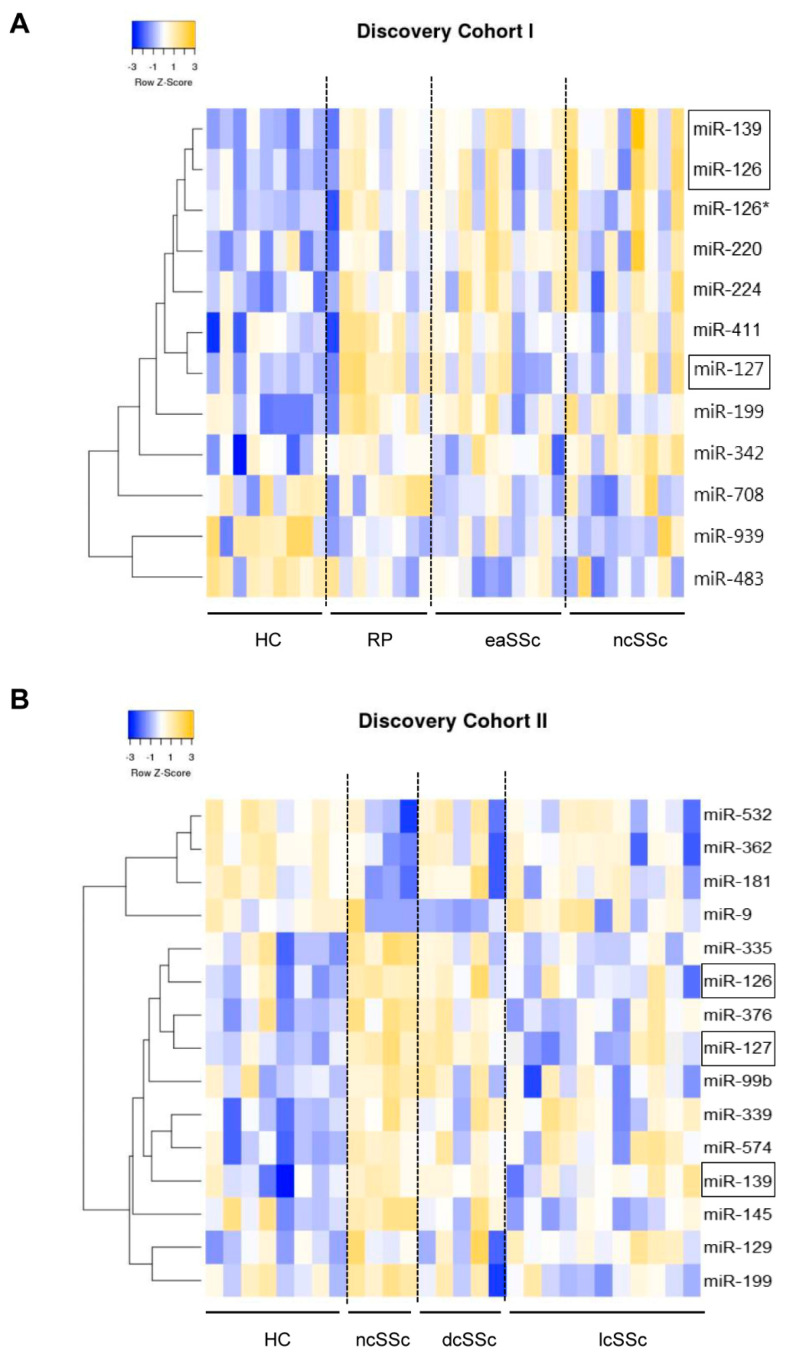
Differentially expressed miRNAs in circulating plasmacytoid dendritic cells of preclinical and definite SSc patients. Two independent experiments were performed for microRNA profiling in RNA extracted from pDCs of two different SSc cohorts using the OpenArray platform in discovery cohort I (HC = 9, RP = 8, eaSSc = 10, ncSSc = 9). (**A**) and discovery cohort II (HC = 8, ncSSc = 4, dcSSc = 5, lcSSc = 11); (**B**). Every donor was screened for 758 miRNAs, and no technical replicates were used. Data were normalized using ExpressionSuite Software (independent t-test, uncorrected *p*-value < 0.05). The expression of each miRNA was calculated as Fold Change (FC) as compared to the healthy control group. MiRNAs presented in heatmaps were differentially expressed with FC of ≥2 or ≤0.5 and a *p*-value of <0.05 in at least one patient group compared to healthy control group. Data are presented on the heatmaps as row z-scores computed from log transformed FC using hierarchical clustering with average linkage. The differentially expressed miRNAs in both cohorts are marked within boxes. HC, healthy controls; RP, Raynaud’s phenomenon; eaSSc, early SSc; ncSSc, non-cutaneous SSc; dcSSc, diffuse cutaneous SSc; lcSSc, limited cutaneous SSc. * indicates *p*-value < 0.05.

**Figure 3:**
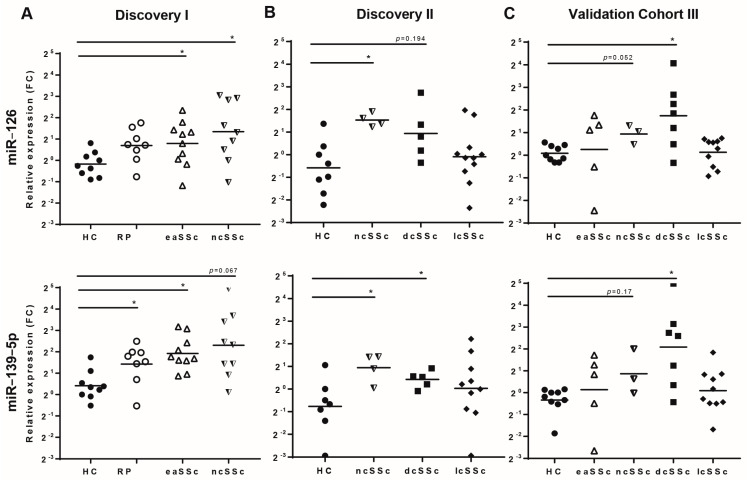
MiR-126 and miR-139-5p are upregulated in SSc patients. The expression of miR-126 and miR-139-5p were analyzed by using the OpenArray platform in the discovery cohort I (HC = 9, RP = 8, eaSSc = 10, ncSSc = 9) (**A**) and II (HC = 8 ncSSc = 4, dcSSc = 5, lcSSc = 11) (**B**) and in the validation cohort (HC = 9, eaSSc = 5, ncSSc = 3, dcSSc = 7, lcSSc = 10) (**C**). The three independent cohorts were screened for data validation, and no technical replicates were used. Data were normalized using ExpressionSuite and ThermoFisher cloud software (independent t-test, uncorrected *p*-value < 0.05). The expression of each miRNA was calculated as Fold Change (FC) as compared to the healthy control group. Data presented as geometric mean (mid-line). HC, healthy controls; eaSSc, early SSc; ncSSc, non-cutaneous SSc; dcSSc, diffuse cutaneous SSc; lcSSc, limited cutaneous SSc, * indicates *p*-value < 0.05.

In discovery cohort I, out of a total of 758, 12 miRNAs were differentially expressed in at least one group of patients compared to healthy controls (Figure 2A and Supplementary Table S1. Fifteen miRNAs were found to be differentially expressed in discovery cohort II (Figure 2B and Supplementary Table S2). Strikingly, miR-126, miR-127, and miR-139-5p were altered in patients with early onset as well as in definite SSc patients in both discovery cohorts I and II (Figure 3A, B), suggesting that these miRNAs are differentially expressed even before the onset of overt skin fibrosis. Further screening of the three commonly altered miRNAs in a third cohort of patients (validation cohort) demonstrated that miR-126 and miR-139-5p were significantly upregulated in dcSSc patients (Figure 3C) and had a trend of upregulation in ncSSc, while the upregulation of miR-127 was not replicated. In light of these results, we opted to further elucidate the potential role of miR-126 and miR-139-5p in pDC dysregulation in the context of SSc pathogenesis.

### 3.3. MiR-126 and miR-139-5p Are Induced by TLR9-Mediated pDC Activation and Correlate with IFN-Responsive Genes 

**Figure 4:**
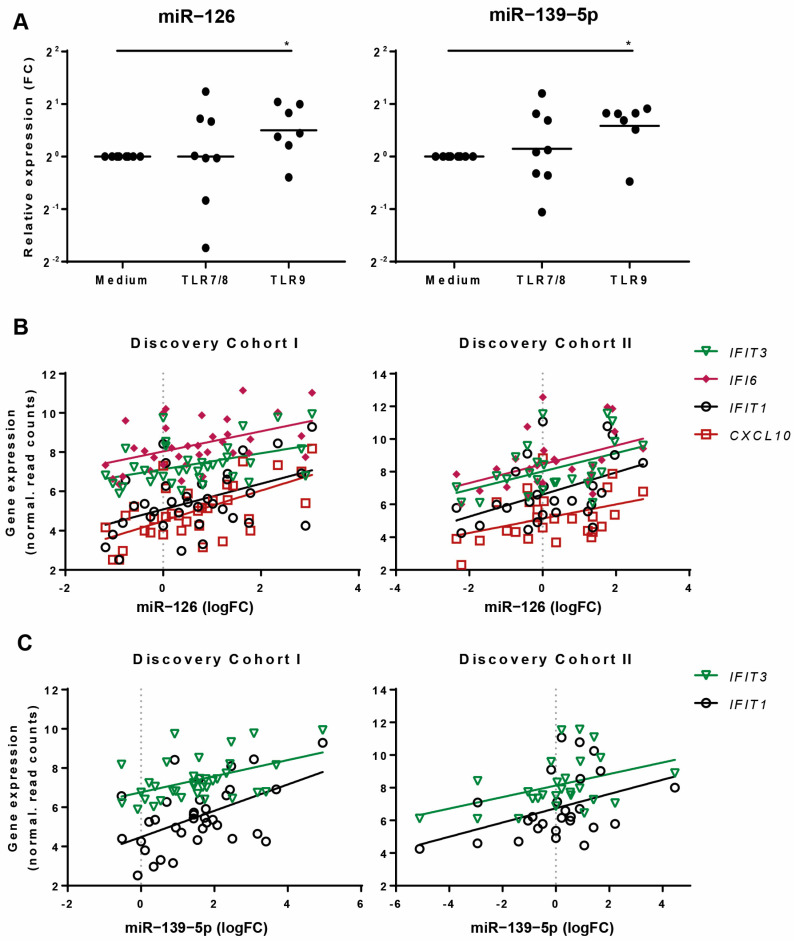
TLR9 stimulation upregulates miR-126 and miR-139-5p expression. (**A**) PDCs from seven to eight independent healthy donors (*n* = 7–8) were stimulated for 24 h with TLR7/8 and TLR9 ligands and the expression of miR-126 and miR-139-5p was assessed by specific qPCR assays. The expression is presented as Fold Change (FC) as compared to the mean expression of the control medium group. Data were normalized to U6 snRNA expression and presented as geometric mean (mid-line) (*p* < 0.05, Wilcoxon signed-rank test to compare each sample to its paired unstimulated condition). * indicates *p*-value < 0.05. (**B**) Correlation of miR-126 with IFN-responsive genes as measured by RNA sequencing in discovery cohort I and II (discovery cohort I: *IFTIT3* r = 0.378 *p* = 0.025, *IFI6* r = 0.468 *p* = 0.005, *IFIT1* r = 0.397 *p* = 0.018 and *CXCL10* r = 0.558 *p* = 0.0005 and discovery cohort II; *IFTIT3* r = 0.444 *p* = 0.023, *IFI6* r = 0.482 *p* = 0.013, *IFIT1* r = 0.471 *p* = 0.015 and *CXCL10* r = 0.397 *p* = 0.045) and (**C**) Correlation of miR-139-5p with IFN-responsive genes as measured by RNA sequencing in discovery cohort I and II (discovery cohort I: *IFIT3* r = 0.403 *p* = 0.016, *IFIT1* r = 0.436 *p* = 0.009 and discovery cohort II *IFIT3* r = 0.411 *p* = 0.037, *IFIT1* r = 0.416 *p* = 0.034). Only IFN genes significantly correlated in both cohorts are reported in **B**,**C** (Spearman’s correlation *p* < 0.05).

To investigate whether the activation status of pDCs in SSc is associated with the aberrant expression of miR-126 and miR-139-5p, we analyzed their expression after the activation of healthy pDCs with TLR7/8 and TLR9 ligands (R848 and CpG-C, respectively) for 24 h. TLR9 stimulation significantly induced the expression of both miR-126 and miR-139-5p, while TLR7/8 did not (Figure 4A). As TLR9 stimulation results in a strong activation of IFN-dependent genes in pDCs, we verified whether the expression of this class of genes was associated with miR-126 and miR-139-5p in SSc patients by correlating miRNA and RNA sequencing data. Interestingly, the expression levels of IFN-induced genes *IFTIT3*, *IFI6*, *IFIT1,* and *CXCL10* levels were positively correlated with miR-126 levels in discovery cohorts I (*IFTIT3* r = 0.378 *p* = 0.025, *IFI6* r = 0.468 *p* = 0.005, *IFIT1* r = 0.397 *p* = 0.018, and *CXCL10* r = 0.558 *p* = 0.0005), as well as in discovery cohort II (*IFTIT3* r = 0.444 *p* = 0.023, *IFI6* r = 0.482 *p* = 0.013, *IFIT1* r = 0.471 *p* = 0.015, and *CXCL10* r = 0.397 *p* = 0.045) (Figure 4B). *IFIT3* and *IFIT1* were also significantly positively correlated with miR-139-5p expression (discovery cohort I: *IFIT3* r = 0.403 *p* = 0.016, *IFIT1* r = 0.436 *p* = 0.009, discovery cohort II *IFIT3* r = 0.411 *p* = 0.037, *IFIT1* r = 0.416 *p* = 0.034) (Figure 4C). These results demonstrate that miR-126 and miR-139-5p are induced in pDCs by an IFN-inducing stimulus and suggest their implication in the IFN signature evident in these cells in SSc patients. 

### 3.4. MiR-126 and miR-139-5p Target Genes Are Enriched in Pathways Related to SSc Pathogenesis

In order to identify the specific pathways targeted by the synergic upregulation of miR-139-5p and miR-126 in SSc, we retrieved their predicted and validated targets and performed a pathway enrichment analysis (Supplementary Figure S1). Enrichment results indicated that miR-126 and miR-139-5p targets are implicated in numerous biological processes that have been previously linked to SSc pathogenesis and/or to pDC biology. The most relevant include Platelet-derived growth factor (PDGF) pathway, Insulin-like growth factor (IGF) pathway, and vascular endothelial growth factor (VEGF) pathway and interleukin signaling (Supplementary Figure S1). Thus, this analysis further substantiated the potential involvement of these miRNAs in SSc-pDC dysregulation and supported additional investigations. 

### 3.5. Proteomics Analysis Identified USP24 as Direct Target of miR-139-5p

**Figure 5:**
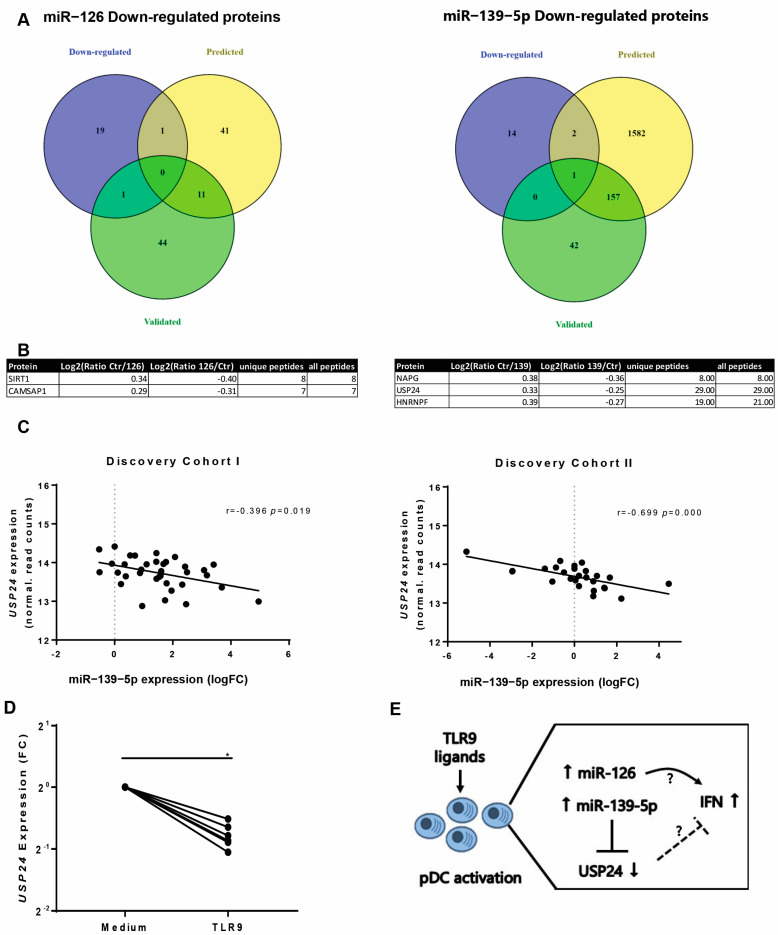
Proteomic analysis to identify the direct targets of miR-126 and miR-139-5p. Stable isotope labeling of amino acids (SILAC) followed by proteomic analysis was performed to identify the targets of miR-126 and miR-139-5p. Only protein targets modulated (log2(ratio miRNA/Ctr) < −0.25 and log2(ratio Ctr/miRNA) > 0.25) in two different biological and technical replicates were taken into consideration. (**A**) Intersection between the down-regulated targets from proteomics analysis (purple circle), the pure predictions of the miRNA in at least three bioinformatic softwares (yellow circle), and the experimental supported or validated targets of the miRNAs (green circle). (**B**) Table depicting the direct protein targets of miR-126 and miR-139-5p filtered out from the intersection of proteomics and predicted and validated targets. (**C**) Correlation of the USP24 gene expression with miR-139-5p in discovery cohort I and in discovery cohort II (Spearman’s correlation *p* < 0.05). (**D**) PDCs of six independent healthy controls (*n* = 6) were stimulated for 24 h with Toll-like receptor 9 (TLR9) ligand, and the expression of USP24 was assessed by single qPCR. The expression is presented as Fold Change (FC) as compared to the mean expression of the control medium group. Data were normalized to the expression of *GUSB* and analyzed with the Wilcoxon signed-rank test (*p* < 0.05). * indicates *p*-value < 0.05. (**E**) Hypothesis model: Putative functional implication of dysregulated miRNAs in SSc pDCs. The chronic activation of pDCs in SSc patients, possibly due to either endogenous or exogenous ligands, leads to an increase of miR-126, which can functionally support IFN response. In parallel, pDC activation promotes also the upregulation of miR-139-5p, which in turn targets USP24, a potential inhibitor of IFN response, thus finally leading to a further potentiation of this pathway.

Despite the fact that miR-126 has been previously linked to pDC activation and IFN response [19], the regulatory network controlled by miR-126 and miR-139-5p has not been extensively investigated so far. Therefore, stable isotope labeling of amino acids (SILAC) followed by proteomic analysis was exploited to further expand the knowledge on direct targets of miR-126 and miR-139-5p. Protein targets were identified in two independent SILAC experiments for each miRNA in HEK cells. A total of 48 and 30 proteins were reproducibly dysregulated after miR-126 and miR-139-5p overexpression respectively, in both experiments. Of these, 38 proteins were downregulated either after miR-126 or miR-139-5p overexpression. To identify the direct targets, from the downregulated proteins, we selected those targets whose coding genes carry complementary seeds for these miRNAs (Figure 5A). Using this approach, we discovered CAMSAP1 as a novel target of miR-126, and NAPG, USP24 as novel targets of miR-139-5p, while we confirmed SIRT1 and HNRNPF as validated targets from the literature of miR-126 and miR-139-5p, respectively (Figure 5B). 

To further substantiate the regulation of these targets by miR-126 and miR-139-5p also in pDCs from SSc patients, we studied the association between the expression of each miRNAs and their respective identified target genes in both discovery SSc cohorts (Supplementary Table S3). The lack of correlation between *CAMSAP1* and *SIRT1* with the expression levels of miR-126 in pDCs, and *NAPG* and *HNRNPF* with the expression levels of miR-139-5p did not confirm the association of these miRNAs with these targets in pDCs of SSc patients. On the contrary, the expression of *USP24* in pDCs was significantly inversely correlated with miR-139-5p levels in both discovery cohort I and II (Figure 5C), corroborating that miR-139-5p targets the USP24 gene and protein expression, both in vitro and ex vivo in pDCs from SSc patients. Moreover, *USP24* was downregulated in pDCs stimulated with a TLR9 agonist (Figure 5D), which is consistent with the evidence that miR-139-5p is induced upon TLR9 stimulation. Overall, these results point at a potential regulatory network involving miR-139-5p, USP24, TLR9-mediated activation, and the IFN-response in pDCs of SSc patients.

## 4. Discussion

Compelling evidence has demonstrated the role of pDCs in SSc pathogenesis [5,6], and it showed that the depletion of pDCs ameliorates skin fibrosis in a mouse model of scleroderma [6]. As the type I IFN signature is evident in SSc patients from the early stages of disease onwards [4], one could hypothesize that pDCs, the major type I IFN-producing cells, might be involved in the onset of the disease. To investigate potential molecular pathways responsible of pDC dysregulation, in the present study, we performed an extensive miRNA screening in pDCs of early preclinical and definite SSc patients. MiRNA profiling revealed that the miRNA signature was altered in pDCs of SSc patients already at early stages of the disease. Of note, miR-126 and miR-139-5p were upregulated in the preclinical SSc patients, and they displayed aberrant expression also in later stages of the disease, indicating that these miRNAs might be associated with the alterations of pDC that underpin SSc development. Moreover, pathway enrichment analysis of miR-126 and miR-139-5p targets demonstrated that these miRNAs regulate pathways associated to SSc pathogenesis, such as IGF, VEGF, and PDGF signaling, further supporting their role in SSc. In order to unravel the molecular mechanisms targeted by these miRNAs in SSc pDCs, we studied multiple layers of analysis, namely miRNome, transcriptome, proteome, in silico prediction, and functional in vitro analysis. 

It is known that TLR activation can convey signaling modulating miRNA expression [20,21,22] and that TLR-responsive miRNAs are tightly interconnected with the regulation of innate immune responses [23]. In line with this, we demonstrate here that pDC activation via TLR9 stimulation induced the expression of both miR-126 and miR-139-5p, similarly to what is observed in pDCs of SSc patients, suggesting that endogenous TLR9 ligands are implicated in the upregulation of these miRNAs in SSc patients. Moreover, the levels of miR-126 and miR-139-5p were positively correlated with the expression of IFN-responsive genes in patients, suggesting a role of these miRNAs in IFN-signaling and immune dysregulation in SSc pathogenesis. This hypothesis is in line with previous studies of Agudo et al. [19], showing that in pDCs, miR-126 controls the innate response to pathogen-associated nucleic acids by regulating interferon production. In addition, miR-126 is also dysregulated in SLE, which is another systemic autoimmune disease [24] that similarly to SSc is characterized by type I IFN-inducible gene expression signature [25,26]. Additionally, miR-126 has been described to modulate PI3K/AKT/mTOR and VEGF signaling [19,27], which are pathways that have been previously shown to be dysregulated in SSc pathogenesis [28,29]. In light of these observations, it could be suggested that miR-126 mediates pDC interferon response also in SSc patients even at the early disease stages, but further research should be performed to validate this hypothesis.

Contrary to miR-126, miR-139-5p has not been linked to pDC biology or autoimmunity so far, while it has been mostly described to be dysregulated in different types of cancer [30]. Several studies have demonstrated that miR-139-5p inhibits cell proliferation, induces apoptosis, and leads to increased oxidative stress [30,31,32,33]. Given that pDCs are reduced in circulation in SSc [9] and that oxidative stress has been associated with disease pathogenesis [34], high levels of miR-139-5p might be involved in cell homeostasis also in pDCs of SSc patients. 

By applying a proteomic approach, we showed that miR-139-5p suppressed the expression of a deubiquitinating enzyme, USP24. Despite the limitation of the proteomic approach in the HEK cell line, we could confirm USP24 involvement in pDCs by the inverse correlation of miR-139-5p levels and USP24 gene expression in pDCs isolated from SSc patients. These results indicated that miR-139-5p regulated USP24 expression not only in vitro but also in patients. Interestingly, multiple deubiquitinating enzymes, such as *USP3*, *USP38* or *DUBA*, negatively regulate type I IFN response [35,36,37,38], and dysfunction of the ubiquitin pathway has been previously linked to autoimmunity [39]. In addition, it is known that ubiquitination can modulate pattern-recognition receptors response [35], and particularly, *USP24* has been reported to be a cofactor required for TLR7/9 signaling [40]. In line with these reports, we also demonstrated that *USP24* is downregulated upon TLR9 stimulation. Even though our study did not provide an in-depth characterization of the mechanism underlying the TLR9–USP24–IFN axis, we postulate that the activation of pDCs in SSc patients could lead to higher miR-139-5p levels, which in turn could fine-tune USP24 expression and thereby possibly mediate IFN response. Nevertheless, the effects of miR-139-5p inhibition on the restoration of its target genes should be examined in upcoming studies both in vitro and in vivo to prove this hypothesis. Additionally, USP24 dysfunction should be evaluated further on SSc, as well as its role in type I IFN production, to understand whether it functions similarly to the other aforementioned deubiquitinating enzymes.

Overall, we show that miR-126 and miR-139-5p are increased in pDCs from patients with SSc from the earliest stages of disease onwards. A unique approach, combining high-dimensional data together with experimental functional experiments and previously reported data, correlated the induction of these miRNAs with relevant pathways crucial for SSc pathogenesis and pDC biology. Above all, both miRNAs conveyed into the regulation of pDC activation via TLR9-mediated response and IFN signaling, which is one of the most evident hallmarks of SSc. Hence, we believe that the elevated levels of miR-126 and miR-139-5p might reflect the activation of circulating pDCs of SSc patients. Considering that the type I IFN signature is present already in early SSc patients before the appearance of overt skin fibrosis [4], further research should be conducted to explore the exact mechanism underlying the role of these miRNAs in SSc and to evaluate the potential therapeutic benefit of modulating their expression in patients.

## Figures and Tables

**Table 1:** Demographics and clinical characteristics of the donors included in the study.

	Discovery Cohort I				Discovery Cohort II				Cohort III: Validation Cohort				
Group (*n*)	HC(9)	RP(8)	eaSSc(10)	ncSSc(9)	HC (8)	ncSSC(4)	dcSSc(5)	lcSSc(11)	HC(9)	eaSSc(5)	ncSSc(3)	dcSSc(7)	lcSSc(10)
Age (yr.)	32 (30–43)	44 (33–67)	53 (46–55)	58 (49–62)	54 (53–61)	43 (35–52)	63 (57–65)	58 (53–67)	53 (46–55)	37 (27–48)	51 (45–55)	46 (35–55)	63 (50–67)
Female (*n*, %)	8 (89%)	8 (100%)	7(78%)	9 (100%)	6 (75%)	3 (75%)	2 (40%)	8 (73%)	7(78%)	4(80%)	3(100%)	4(57%)	9(90%)
ANA (*n* pos, %)	-	3 (38%)	9(90%)	9 (100%)	-	4 (100%)	5 (100%)	10 (91%)	-	3(60%)	2(67%)	6(86%)	9(90%)
ACA (*n* pos %)	-	0	6(60%)	6 (67%)	-	2 (50%)	2 (40%)	6 (55%)	-	2(40%)	1(33%)	1(14%)	9(90%)
Scl70 (*n* pos, %)	-	0	2(20%)	1 (11%)	-	2 (50%)	2 (40%)	2 (18%)	-	0	1(33%)	3(43%)	3(30%)
mRSS	-	0	0	0	-	0	20 (10–30)	7 (3–9)	-	0	0	16 (8–20)	9 (3–15)
ILD	-	0	0	0	-	1	4	2	-	0	1 (33%)	1 (14%)	4 (40%)
Disease Duration	-	-	-	-	-	3 (3–5)	16 (8–26)	4 (3–12)	-	-	3 (2–4)	3 (1–15)	10 (3–23)

Values depict the number of patients and the median for each parameter (Interquartile Range (IQR)), if not otherwise indicated. Yr., years; ANA, antinuclear antibodies; pos, positivity; ACA, anticentromere antibodies; Scl70, antitopoisomerase antibodies; mRSS, modified Rodnan Skin score; ILD, Interstitial Lung disease; HC, healthy controls; RP, Raynaud’s Phenomenon; eaSSc, early SSc; ncSSc, non-cutaneous SSc; dcSSc, diffuse cutaneous SSc; lcSSc, limited cutaneous SSc.

## Data Availability

All relevant raw data from the data presented in the manuscript or the supplementary figures and tables are available by the authors of the study upon request.

## Supplementary Materials

The following are available online at https://www.mdpi.com/2077-0383/10/3/491/s1, Figure S1: Enriched signaling pathways of miR-126 and miR-139-5p target genes, Table S1: MiRNAs differentially expressed in circulating plasmacytoid dendritic cells of preclinical and non-cutaneous SSc patients (discovery cohort I), Table S2: MiRNAs differentially expressed in circulating plasmacytoid dendritic cells of definite SSc patients (discovery cohort II), Table S3: The identified targets of miR-126 and miR-139-5p by proteomics were correlated with the levels of the miRNAs and their target genes in the same individuals participating in the study.
