# Supplementary material for: Implication of miR-126 and miR-139-5p in Plasmacytoid Dendritic Cell Dysregulation in Systemic Sclerosis
Source: J Clin Med. 2021 Jan 30;10(3):491. doi: 10.3390/jcm10030491 (PMC7866506; doi:10.3390/jcm10030491)
Supplement: Supplementary file 1 [file jcm-10-00491-s001.pdf]

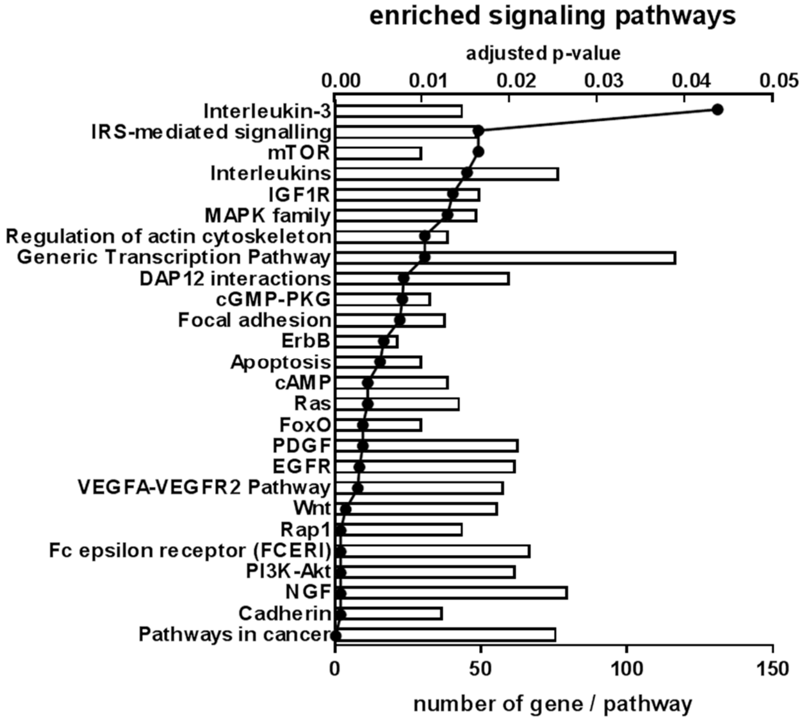

Figure S1. Enriched Signaling Pathways of miR-126 and miR-139-5p target genes. Pathway enrichment was performed on the predicted and validated targets of miR-139-5p and miR-126 to assess their potential synergic effect in SSc. For the pathway enrichment analysis we retrieved all the predicted and validated targets of miR-126 and miR139-5p. MirWalk 2.0 database (http://zmf.umm.uni-heidelberg.de/apps/zmf/mirwalk2 /custom.html) was used to retrieve the predicted targets from 5 prediction software (Miranda, miRDB, Pictar2, PITA, RNA22, Targetscan). We also used miRWalk database to retrieve all the validated target genes of miR-126 and miR-139-5p. ToppGene Suite website (https://toppgene.cchmc.org/enrichment.jsp) was used for the pathway enrichment analysis of the aforementioned predicted and validated target genes of miR-126 and miR-139-5p. The boxes represent the number of genes per pathway and the line represent the adjusted p-value.

Table S1. MiRNAs differentially expressed in circulating plasmacytoid dendritic cells of preclinical and non-cutaneous SSc patients (Discovery Cohort I). MicroRNA profiling was performed in RNA extracted from pDCs of SSc patients and healthy controls using the OpenArray platform. The expression of each miRNA was calculated as Fold Change (FC) as compared to the healthy control group. MiRNAs were considered differentially expressed with a FC of ≥ 2 or ≤ 0,5 and a p-value of < 0,05 in at least one patient-group vs healthy controls; RPANA, Raynaud's Phenomenon; eaSSc, early SSc; ncSSc, non-cutaneous SSc.

	**RP**		**eaSSc**		**ncSSc**	
**miRNA**	**FC**	**p value**	**FC**	**p value**	**FC**	**p value**
miR-126	1.828	0.034	**1.955**	**0.026**	**2.873**	**0.028**
miR-139-5p	**2.011**	**0.021**	**2.844**	**0.006**	3.709	0.067
miR-126#	**3.245**	**0.039**	**2.051**	**0.048**	3.697	0.065
miR-939	**0.076**	**0.029**	**0.128**	**0.036**	0.125	0.313
miR-127	**2.816**	**0.021**	1.635	0.088	1.761	0.128
miR-199a-5p	**2.730**	**0.027**	2.107	0.061	2.186	0.077
miR-411	**2.615**	**0.039**	1.971	0.134	1.783	0.226
miR-220	2.747	0.304	**5.201**	**0.013**	4.300	0.114
miR-224	2.261	0.122	**2.930**	**0.043**	2.745	0.060
miR-483-5p	0.272	0.053	**0.060**	**0.005**	0.163	0.462
miR-708	1.063	0.806	**0.487**	**0.048**	0.552	0.571
miR-342-5p	2.449	0.132	1.828	0.268	**4.306**	**0.011**

Table S2. MiRNAs differentially expressed in circulating plasmacytoid dendritic cells of definite SSc patients (Discovery Cohort II). MicroRNA profiling was performed in RNA extracted from pDCs of SSc patients and healthy controls using the OpenArray platform. The expression of each miRNA was calculated as Fold Changes (FC) as compared to the healthy control group. MiRNAs were considered differentially expressed with a FC of ≥ 2 or ≤ 0,5 and a p-value of < 0,05 in at least one patient-group vs healthy controls; ncSSc, non-cutaneous SSc; lcSSc, limited cutaneous SSc; dcSSc, diffuse cutaneous SSc.

	**ncSSc**		**dcSSc**		**lcSSc**	
**miRNA**	**FC**	**p value**	**FC**	**p value**	**FC**	**p value**
<u>miR-126</u>	**4.321**	**0.001**	2.871	0.194	1.410	0.373
<u>miR-139-5p</u>	**4.763**	**0.029**	**3.327**	**0.030**	3.341	0.190
miR-199a-3p	**2.399**	**0.017**	0.796	0.970	0.753	0.370
miR-145	**2.198**	**0.038**	1.196	0.784	0.792	0.295
<u>miR-127</u>	5.293	0.053	**3.491**	**0.033**	0.221	0.600
miR-574-3p	**2.600**	**0.002**	1.950	0.099	**2.020**	**0.010**
miR-335	**3.995**	**0.029**	1.554	0.567	1.119	0.690
miR-99b	**2.051**	**0.044**	1.334	0.412	0.914	0.854
miR-376c	**3.057**	**0.046**	2.015	0.136	1.213	0.760
miR-339-3p	**2.120**	**0.049**	1.647	0.105	1.682	0.005
miR-1291	2.175	0.312	1.317	0.400	**2.066**	**0.020**
miR-362	**0.419**	**0.013**	0.688	0.476	0.655	0.068
miR-181a-2#	**0.330**	**0.026**	0.794	0.917	0.706	0.217
miR-9	0.532	0.899	**0.345**	**0.002**	1.031	0.419
miR-532	0.446	0.097	0.805	0.837	0.681	0.073

Table S3. The identified targets of miR-126 and miR-139-5p by proteomics were correlated with the levels of the miRNAs and their target genes in the same individuals participating in the study.

	**Discovery I**		**Discovery II**	
**Target genes of miR-126**	**r**	**p value**	**r**	**p value**
SIRT1	-0.083	0.635	-0.218	0.284
CAMPSAP1	-0.230	0.183	-0.259	0.202
	**Discovery I**		**Discovery II**	
**Target genes of miR-139-5p**	**r**	**p value**	**r**	**p value**
<u>USP24</u>	**-0.396**	**0.019**	**-0.699**	**0.000**
NAPG	-0.248	0.151	-0.219	0.283
HNRPF	-0.137	0.433	0.257	0.205
